# The Silent Intruder: A Case Report of an Incidentally Discovered Solitary Fibrous Tumor of the Lung

**DOI:** 10.7759/cureus.113019

**Published:** 2026-07-20

**Authors:** Nisat Tasnim, Danny Thai, Sadia Bari, Montasin Rezwan, Tanzina Nowrin

**Affiliations:** 1 Internal Medicine, Flushing Hospital Medical Center, New York, USA; 2 Clinical Sciences, St. George’s University School of Medicine, St. George's, GRD; 3 Internal Medicine, Capital Health Medical Center Hopewell, Pennington, USA

**Keywords:** case report, cd34, immunohistochemistry, intrapulmonary, mesenchymal neoplasm, nab2-stat6 fusion, solitary fibrous tumor, stat6, surgical resection, video-assisted thoracoscopic surgery

## Abstract

Solitary fibrous tumors (SFTs) are rare fibroblastic mesenchymal neoplasms with a predominantly benign, indolent course and low metastatic potential. Although SFTs most commonly arise within the pleura of the thoracic cavity, primary intrapulmonary origin, wherein the tumor arises within the lung parenchyma itself rather than the pleural surface, represents an exceptionally rare and diagnostically challenging clinical entity. Here, we present the case of an 80-year-old man with an incidentally discovered SFT in the left lung during an active tuberculosis (TB) rule-out due to a positive QuantiFERON-TB Gold test performed as part of a mandatory screening protocol upon enrollment as a new nursing home resident. The review of systems was negative, and the physical examination was unremarkable except for decreased breath sounds in the left lower lung field. Chest X-ray demonstrated an ovoid left lung mass measuring approximately 14 cm in diameter, subsequently confirmed on CT as a 12.5 × 10.4 × 6.5 cm well-demarcated soft tissue tumor in the left lower lobe. The diagnosis was confirmed by histopathologic examination and immunohistochemistry (IHC) of a biopsy specimen from the left lung mass, which demonstrated characteristic spindle cell morphology with STAT6 and CD34 nuclear positivity. The patient was referred to hematology oncology for further management and subsequently underwent complete surgical resection via video-assisted thoracoscopic surgery. This case expands the clinicopathological spectrum of primary intrapulmonary SFTs, demonstrating that atypical presentations may remain clinically silent yet be identified through systematic radiological evaluation. Surgical resection with long-term surveillance remains essential, and prospective multicenter studies are warranted to develop guidelines specific to this rare entity.

## Introduction

SFTs are rare fibroblastic spindle-cell neoplasms that account for approximately 3.7% of visceral and soft tissue sarcomas [1,2]. While SFTs account for approximately 3.7% of visceral and soft tissue sarcomas, soft tissue sarcomas themselves represent fewer than 1% of all adult malignancies, making SFTs extremely uncommon in absolute terms within the general population [1]. Most are benign, with low malignant potential and metastatic potential, and often remain clinically asymptomatic until a sufficient tumor burden compresses adjacent structures [1]. No established environmental risk factors, gender predispositions, or hereditary associations have been identified. SFTs occur across a broad age spectrum, typically affecting patients aged 20-70 years, with peak incidence in patients aged 40-60 years [1].

Within the thoracic cavity, SFTs most commonly arise from the pleural surface, with fewer than 1,000 pleural cases reported in the literature, occurring at an incidence of fewer than three per 100,000 hospital admissions and representing less than 5% of all pleural tumors [3]. Primary intrapulmonary SFTs, arising within the lung parenchyma rather than the pleural surface, are considerably rarer and most often discovered incidentally on chest CT as well-demarcated soft tissue masses, typically measuring between 1 and 10 cm in diameter [2,4,5]. Tumors larger than 10 cm carry an increased risk of malignant behavior, and larger tumors have been associated with more aggressive NAB2-STAT6 fusion variants [3,6].

The molecular hallmark of SFT is a chromosomal 12q13 rearrangement resulting in a NAB2-STAT6 gene fusion, which disrupts normal transcriptional repression and constitutively activates fibroblastic proliferation and tumor growth, in part through overexpression of vascular endothelial growth factor (VEGF) [1,6,7]. At least 12 distinct NAB2-STAT6 fusion variants have been characterized, with certain variants, particularly NAB2 exon 4-STAT6 exon 2, associated with older patient age at presentation and larger thoracic tumors [6]. Diagnosis is confirmed through histopathological examination and immunohistochemistry (IHC), with nuclear STAT6 positivity representing the most sensitive and specific diagnostic marker, frequently accompanied by CD34 expression [1,6].

Given the exceptional rarity of primary intrapulmonary SFTs, the existing literature is largely confined to small case series and isolated case reports, leaving significant gaps in the understanding of their natural history, growth kinetics, and optimal management [2,4]. We herein report a rare case of an 80-year-old man with a 12.5 × 10.4 × 6.5 cm benign primary intrapulmonary SFT of the left lung, notable for its occurrence beyond the typical age range, its primary intrapulmonary rather than pleural origin, its rapid growth trajectory, and the patient's complete clinical silence despite exceptional tumor burden. This case contributes to the limited body of literature on intrapulmonary SFTs and underscores the critical role of incidental imaging findings in the early detection of this diagnostically challenging neoplasm.

## Case presentation

An 80-year-old man with a past medical history of prostate cancer and benign prostatic hyperplasia was admitted to the hospital to rule out active tuberculosis (TB) after his QuantiFERON-TB Gold test was positive. The QuantiFERON-TB Gold test was performed as part of a mandatory TB screening protocol upon the patient's enrollment as a new nursing home resident; a positive result prompted hospital admission for active TB rule-out. The patient had no prior surgical history. Details of prior prostate cancer treatment were unavailable in the accessible clinical records. Family history was unremarkable, with no known history of malignancy. Social history was notable for active marijuana use for more than five years and a history of cocaine and heroin abuse. The patient denied any symptoms, and blood work was unremarkable (Table 1). His physical examination was within normal limits except for decreased breath sounds over the left lower lung fields.

**Table 1:** Complete blood count and comprehensive metabolic panel results

Laboratory parameter	Observed value	Reference range	Units
Complete blood count with platelet and differential			
White blood cells	8	3.8-11.0	x10^3/µL
Red blood cells	4.42	4.20-5.80	x10^6/µL
Hemoglobin	12.1	13.2-17.1	g/dL
Hematocrit	38.1	38.5-50.0	%
Mean corpuscular volume	86.2	80.0-100.0	fL
Mean corpuscular hemoglobin	27.4	27.0-33.0	pg
Mean corpuscular hemoglobin concentration	31.8	32.0-36.0	g/dL
Red cell distribution width	15.1	11.0-15.0	%
Mean platelet volume	11	9.0-12.2	fL
Platelets	263	140-400	x10^3/µL
Neutrophils auto	67.4	40.0-70.0	%
Lymphocytes auto	20.7	18.0-42.0	%
Monocytes auto	8.4	4.0-11.0	%
Eosinophils auto	2.7	0.0-5.0	%
Basophils auto	0.8	0.0-2.0	%
Neutrophils absolute	5.3	2.0-4.8	x10^3/µL
Lymphocytes absolute	1.7	1.1-3.2	x10^3/µL
Monocytes absolute	0.7	0.1-0.8	x10^3/µL
Eosinophils absolute	0.2	0.0-0.4	x10^3/µL
Basophils absolute	0.1	0.0-0.1	x10^3/µL
Comprehensive metabolic panel			
Glucose	110	70-100	mg/dL
Blood urea nitrogen	18	Sep-23	mg/dL
Creatinine	0.7	0.7-1.3	mg/dL
Sodium	139	137-145	mmol/L
Potassium	3.9	3.5-5.1	mmol/L
Chloride	103	98-107	mmol/L
Carbon dioxide	23	22-30	mmol/L
Calcium	9.2	8.6-10.2	mg/dL
Anion gap	13	7.0-16.0	mmol/L
Protein, total	6.5	6.4-8.2	g/dL
Albumin	3.7	3.5-5.0	g/dL
Bilirubin, total	0.4	0.2-1.2	mg/dL
Alanine aminotransferase	32	0-49	U/L
Aspartate aminotransferase	33	17-59	U/L
Alkaline phosphatase	110	38-126	U/L

**Figure 1:**
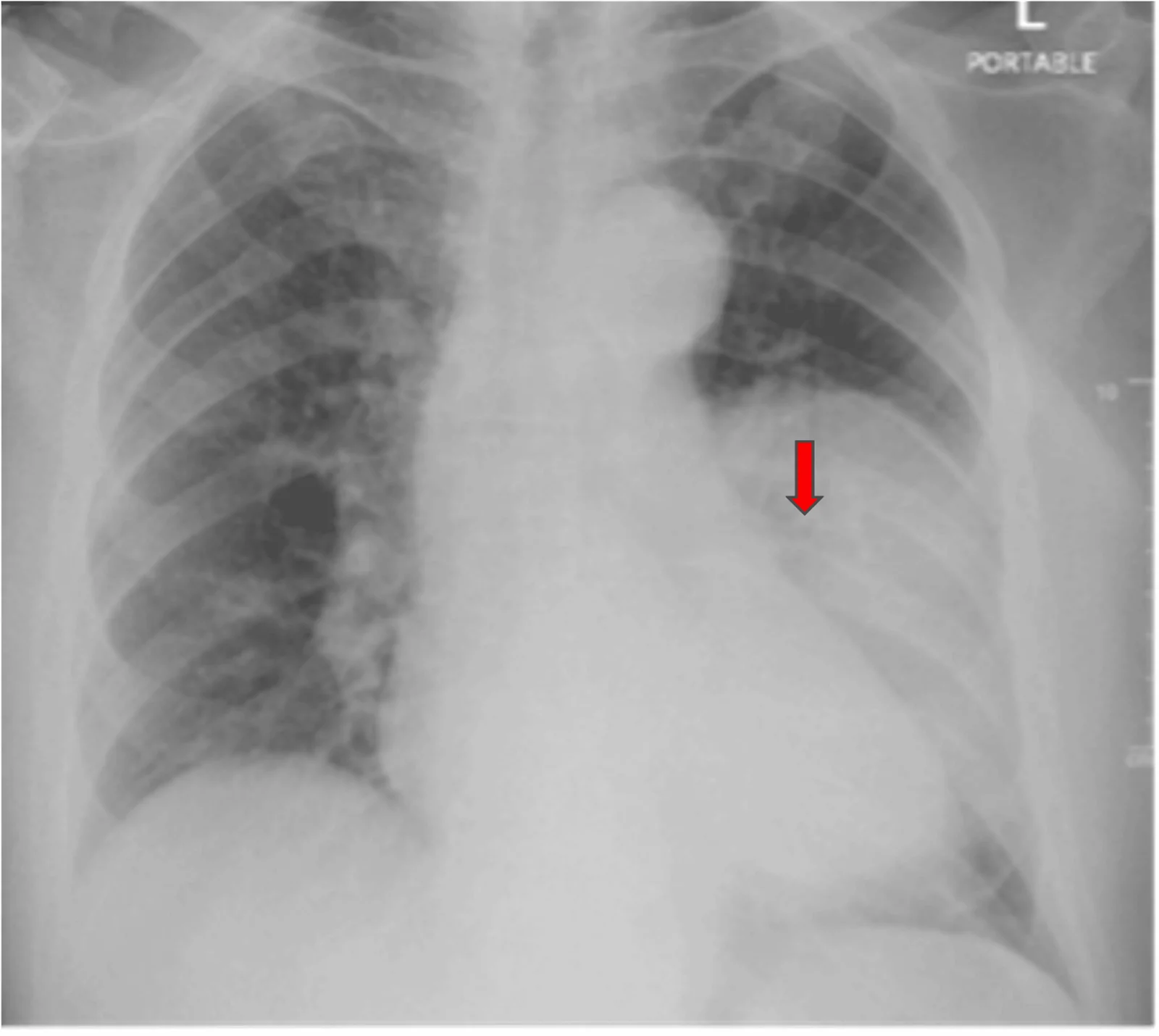
Posteroanterior chest X-ray demonstrating a large ovoid left lung mass measuring approximately 14 cm in craniocaudal diameter consistent with an intrapulmonary soft tissue tumor

A chest X-ray obtained approximately three years before presentation was negative for masses. A follow-up chest X-ray obtained approximately two years before presentation demonstrated a 5.8 × 5.8 cm perihilar density. As part of the infectious disease evaluation, chest radiography was obtained initially, followed by CT to further characterize the abnormality. A chest X-ray obtained at presentation (Day 0) demonstrated an ovoid mass in the left lung measuring approximately 14 cm in diameter (Figure 1).

Serial imaging documented tumor progression from an undetectable lesion approximately 3 years before presentation to a 5.8 × 5.8 cm perihilar density approximately two years before presentation, and ultimately to a 12.5 × 10.4 × 6.5 cm mass on CT at presentation (Day 0), representing nearly a threefold increase in size within less than two years, a growth trajectory atypical for a benign mesenchymal neoplasm.

**Figure 2:**
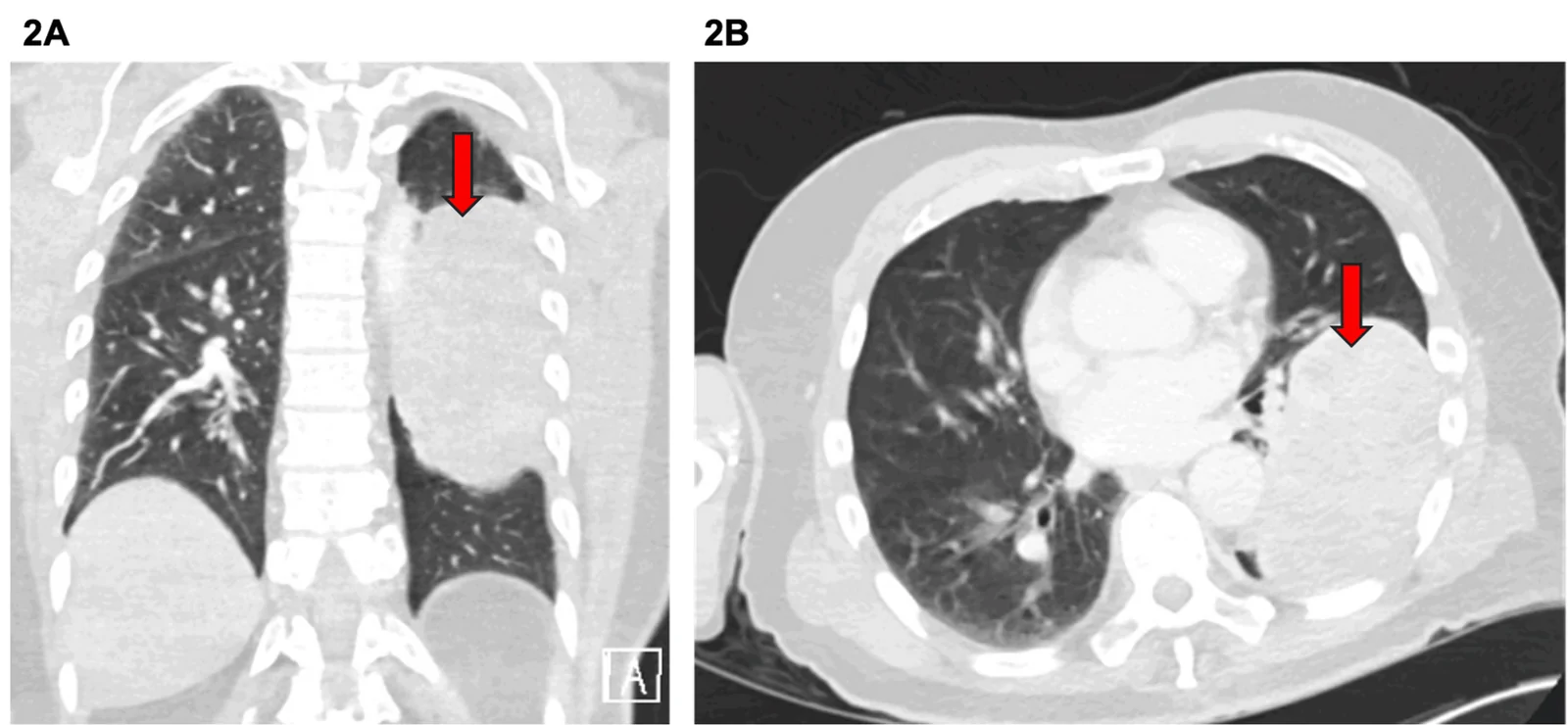
CT of the chest demonstrating a 12.5 × 10.4 × 6.5 cm well-demarcated soft tissue tumor in the left lower lobe without pleural effusion, pleural thickening, or pneumothorax, with incidental peripheral filling defects within the pulmonary arterial branches of the left upper lobe and right lower lobe suggestive of pulmonary embolism

A chest CT performed on Day 0 revealed a 12.5 × 10.4 × 6.5 cm well-demarcated soft tissue tumor in the left lower lobe, with no evidence of pleural effusion, pleural thickening, or pneumothorax (Figure 2A-2B). Incidentally, small peripheral filling defects were identified within the pulmonary arterial branches of the left upper lobe and right lower lobe, findings suggestive of pulmonary embolism (Figure 2A-2B).

**Figure 3:**
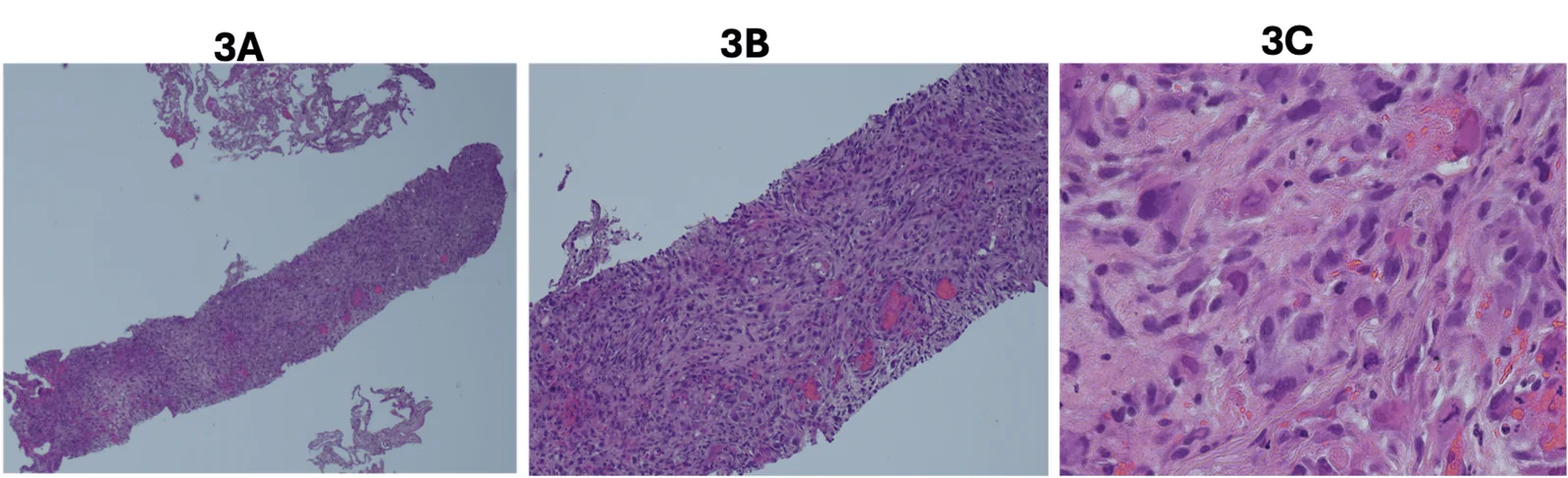
Biopsy sections demonstrating neoplastic spindle cell lesions in a dense fibrous background with a network of branching, dilated vessels. No significant nuclear pleomorphism or necrosis is identified

Hematology/oncology was consulted, and a CT-guided core needle biopsy of the left lung mass was performed. Pathology findings were consistent with an SFT. Histopathological examination revealed neoplastic spindle cell lesions arranged haphazardly within a dense fibrous background, exhibiting a characteristic network of branching, dilated hemangiopericytoma-like vessels (Figure 3A-3C). No significant nuclear pleomorphism, tumor necrosis, or lymphovascular invasion was identified, and markedly low mitotic activity was noted, collectively supporting a benign histopathological diagnosis despite the exceptional tumor size.

IHC analysis demonstrated positivity for the following markers: CD34, STAT6 (nuclear), Vimentin, smooth muscle actin, and Desmin. The neoplastic cells were negative for AE1/AE3, CD68, CD117, Desmin, DOG1, EMA, ER, Factor XIIIa, S100, and SOX10. The Ki-67 proliferation index was 1-4%, consistent with a low-grade lesion. The combination of STAT6 positivity and CD34 expression, alongside negativity for epithelial, neural, and smooth muscle markers, was consistent with the characteristic immunohistochemical profile of SFT.

The patient was initiated on therapeutic-dose anticoagulation for suspected pulmonary embolism based on CT findings and discharged with scheduled outpatient oncology follow-up. He subsequently underwent video-assisted thoracoscopic surgery (VATS) for complete surgical resection of the tumor mass, representing an appropriate and minimally invasive approach for this intrapulmonary tumor. The patient tolerated the VATS resection without immediate postoperative complications; however, long-term follow-up data were not available in the accessible clinical records at the time of manuscript preparation, representing a limitation of this report. Long-term clinical surveillance has been recommended given the well-documented potential for late recurrence in SFTs.

## Discussion

This case presents several clinically noteworthy features that collectively distinguish it from previously reported SFT cases. The patient's age at presentation (80 years) exceeds the reported peak incidence range of the fifth and sixth decades of life [1]. Furthermore, the tumor's primary intrapulmonary origin, rather than the far more common pleural surface, places this case in an exceptionally rare subset of SFT presentations [2,4]. Notably, the patient remained entirely asymptomatic despite measuring more than 12 cm in greatest dimension, with decreased breath sounds on auscultation being the sole clinical finding. We acknowledge that decreased breath sounds on examination constitute an objective clinical sign; however, the patient denied any subjective respiratory symptoms, including cough, dyspnea, chest pain, and hemoptysis. The characterization of this presentation as "clinically silent" refers to the complete absence of patient-reported symptoms attributable to the mass, which is clinically remarkable given its exceptional size and consistent with previously reported large SFTs presenting without symptoms [2,4]. This finding is particularly noteworthy given that approximately 40-60% of SFT patients are symptomatic at presentation, typically manifesting cough, chest pain, or dyspnea, while hemoptysis and obstructive pneumonitis represent rare occurrences resulting from direct airway compression [6]. The complete absence of such symptoms in this patient, despite the exceptional tumor burden, is consistent with an expansile, rather than invasive, growth pattern characteristic of benign intrapulmonary SFTs [2,4]. This case therefore exemplifies how primary intrapulmonary SFTs can attain remarkable dimensions in clinical silence, rendering incidental radiological detection the sole means of timely diagnosis.

The rapid growth trajectory observed in this case warrants particular scientific interest. Serial imaging documented tumor progression from an undetectable lesion approximately three years before diagnosis to a 5.8 × 5.8 cm perihilar density approximately two years before diagnosis, and ultimately to a 12.5 × 10.4 × 6.5 cm mass at the time of diagnosis, representing nearly a tripling in size over less than two years. Such growth kinetics are atypical for a benign mesenchymal neoplasm and may reflect an underlying aggressive molecular variant. The molecular hallmark of SFT is a chromosomal 12q13 rearrangement resulting in a NAB2-STAT6 gene fusion [1,6]. This fusion disrupts the normal transcriptional repressor function of NAB2 and constitutively activates STAT6-mediated fibroblastic proliferation, in part through VEGF overexpression [6,7]. At least 12 distinct NAB2-STAT6 fusion variants have been characterized [6]. Among these, the NAB2 exon 4-STAT6 exon 2 (N4S2) variant has been specifically associated with older patient age at presentation and larger thoracic tumors exceeding 10 cm [6]. The clinicopathological profile of our patient, advanced age, exceptional tumor size, and rapid growth, is compatible with, but does not confirm, this fusion variant. Molecular subtyping was not performed in this case and represents an important avenue for future investigation.

Several previously reported cases of primary intrapulmonary SFTs share clinical features with the present case. Alghamdi et al. reported a 24-year-old female with a benign intrapulmonary SFT who underwent successful surgical resection [4]. Ichiki et al. described a 68-year-old male with a pulmonary SFT confirmed by IHC, including CD34 and STAT6 positivity, who was treated with VATS wedge resection [3]. Wang et al. reported a clinicopathological series of 52 intrapulmonary SFT cases with a mean age of onset of 52.7 years, the majority of which were borderline tumors managed with surgical resection [2]. Collectively, these cases reinforce the role of surgical resection as the cornerstone of management, with a generally favorable prognosis in histologically benign tumors.

Despite exceeding the 10 cm threshold for malignant behavior, the histopathological examination revealed no significant nuclear pleomorphism, tumor necrosis, or lymphovascular invasion. Mitotic activity was markedly low, with only rare mitotic figures identified on histopathological examination, and the Ki-67 proliferation index of 1-4% further corroborated the low-grade proliferative nature of this tumor [3,4]. This discordance highlights that tumor size alone is an insufficient predictor of malignant behavior in SFTs [3,4,8].

Formal risk stratification was performed using two validated multiparametric scoring systems. Based on the Demicco risk-scoring system: age ≥55 years (1 point), tumor size 10-15 cm (2 points), mitotic activity <1/10 HPF (0 points), and necrosis <10% (0 points), the patient achieves a Demicco score of 3, placing the tumor in the low-risk category despite its exceptional size (Table 2).

**Table 2:** Application of the Demicco risk-scoring system to the present case Risk category: low risk (score 0-3) The Demicco risk-scoring system stratifies SFTs into three risk categories based on four histopathological and clinical parameters: low risk (scores 0-3), intermediate risk (scores 4-5), and high risk (scores 6-8). A total score of 3 in this patient, driven primarily by advanced age (1 point) and exceptional tumor size (2 points), places this tumor in the low-risk category despite its large size, providing objective support for the benign histological designation. HPF: high-power field, SFTs: solitary fibrous tumors

Parameter	Scoring criteria	Finding in this case	Score assigned
Patient age	<55 years = 0 points; ≥55 years = 1 point	80 years	1
Tumor size	<5 cm = 0 points; 5-10 cm = 1 point; 10-15 cm = 2 points; >15 cm = 3 points	12.5 cm (greatest dimension)	2
Mitotic activity	<1/10 HPF = 0 points; ≥1/10 HPF = 4 points	Rare mitotic figures (<1/10 HPF)	0
Necrosis	<10% = 0 points; ≥10% = 1 point	Absent	0
Total score			3

Complementary application of the Tapias risk-scoring system, which assigns one point each for parietal pleural origin, sessile morphology, tumor size >10 cm, hypercellularity, necrosis or hemorrhage, and mitotic activity ≥4/10 HPF, yields a minimum score of 2 in this patient, driven by tumor size exceeding 10 cm (1 point) and hypercellularity (1 point), also falling below the high-risk threshold of ≥3 (Table 3) [9]. Tumor morphology could not be reliably assessed from the available data, representing a scoring limitation for the Tapias system. It is important to acknowledge that both scoring systems were originally developed and validated for pleural SFTs, and their direct applicability to primary intrapulmonary tumors, as in this case, has not been formally validated. Notwithstanding these caveats, the concordant low-risk designation across both scoring systems provides additional objective support for the benign histological designation. It is consistent with the favorable IHC profile demonstrated in this case. Retained CD34 positivity, combined with the absence of cytokeratin AE1/AE3 expression, further corroborated a benign biological profile, consistent with previously reported markers of low malignant potential in SFTs [3,7].

**Table 3:** Application of the Tapias risk-scoring system to the present case Risk category: low risk (score <3) The Tapias risk-scoring system assigns one point for each of six adverse histopathological features: parietal pleural origin, sessile morphology, tumor size greater than 10 cm, hypercellularity, necrosis or hemorrhage, and mitotic activity of four or more per 10 high-power fields, with a total score of three or greater indicating high risk of recurrence [10]. In this patient, a minimum score of 2 was calculated, driven by tumor size exceeding 10 cm (1 point) and hypercellularity (1 point), while necrosis, hemorrhage, and significant mitotic activity were absent. This score falls below the high-risk threshold of ≥3, providing additional objective support for the benign designation. * The Tapias system was originally developed for pleural SFTs; parietal pleural origin scores 1 point. As this tumor is of primary intrapulmonary origin, this criterion was scored 0, though this parameter may not be directly applicable to intrapulmonary SFTs. † Tumor morphology (sessile vs. pedunculated) was not explicitly documented in the available pathological or radiological reports and could not be reliably scored, representing a data limitation. HPF: high-power field, SFTs: solitary fibrous tumors

Parameter	Adverse criterion (1 point)	Finding in this case	Score assigned
Pleural origin	Parietal pleural origin	Primary intrapulmonary origin (not pleural)	0*
Tumor morphology	Sessile	Not documented in available records	Indeterminate†
Tumor size	>10 cm	12.5 cm (greatest dimension)	1
Hypercellularity	Present	Present (neoplastic cellular spindle cell lesions noted)	1
Necrosis or hemorrhage	Present	Absent	0
Mitotic activity	≥4/10 HPF	Rare mitotic figures (<4/10 HPF)	0
Total score			2 (minimum)

The IHC profile of this tumor demonstrated nuclear STAT6 positivity and CD34 expression, alongside negativity for AE1/AE3, EMA, S100, SOX10, CD117, and DOG1. This profile provided a characteristic immunohistochemical pattern consistent with SFT, effectively excluding synovial sarcoma, schwannoma, malignant peripheral nerve sheath tumor, and gastrointestinal stromal tumor from the differential diagnosis [1,6,7]. Nuclear STAT6 immunoreactivity directly reflects the underlying NAB2-STAT6 gene fusion and is recognized as the most sensitive and specific IHC marker for SFT diagnosis [6,7].

The incidental identification of peripheral pulmonary arterial filling defects on CT, suggestive of pulmonary embolism, represents an additional clinically significant finding. Although pulmonary embolism is not an established paraneoplastic manifestation of SFT, large intrapulmonary tumors may predispose to thromboembolic events through mechanical vascular compression and tumor-associated procoagulant activity, warranting heightened clinical vigilance in similar presentations. In this case, therapeutic-dose anticoagulation was initiated based on CT findings consistent with pulmonary embolism. V/Q scintigraphy was not performed because CT findings were deemed sufficient for clinical decision-making; however, V/Q scintigraphy remains a valuable complementary diagnostic modality for pulmonary embolism, particularly when CT pulmonary angiography findings are inconclusive, or CT is not feasible. Neither of the two well-recognized paraneoplastic syndromes associated with SFTs, hypertrophic osteoarthropathy (Pierre-Marie-Bamberg syndrome), occurring in fewer than 10% of cases, and Doege-Potter syndrome, occurring in fewer than 5% of cases, were observed in this patient, consistent with their overall rarity [1].

Surgical resection with clear margins (R0) remains the cornerstone of treatment for resectable SFTs [10]. This patient underwent VATS, which represents an appropriate and increasingly adopted minimally invasive surgical approach for intrapulmonary tumors [5,10]. For unresectable or recurrent disease, antiangiogenic agents such as pazopanib have demonstrated an overall response rate of 51% in a phase II trial of 36 patients with advanced SFTs [6]. However, cardiovascular screening is warranted prior to initiation, given associated risks of QTc prolongation, hypertension, and cardiomyopathy [11]. Long-term clinical and radiological surveillance remains essential, as SFT recurrence has been documented more than 20 years after initial surgical resection [4,6,10]. This case highlights that established clinicopathological paradigms for SFTs may not reliably apply across all presentations, underscoring the indispensable role of systematic radiological evaluation and rigorous IHC-based diagnosis in identifying this exceptionally rare neoplasm.

## Conclusions

This case contributes to the limited literature on primary intrapulmonary SFTs, documenting an atypical presentation that broadens the recognized clinicopathological spectrum of this rare neoplasm. It further underscores the important role of incidental radiological detection and systematic pathological evaluation in the timely diagnosis of this rare entity. Surgical resection was successfully achieved, and long-term surveillance remains imperative given the well-documented potential for late recurrence.

The primary limitations of this case include the absence of molecular subtyping, incomplete documentation of prior prostate cancer treatment, and unavailability of detailed operative and postoperative follow-up data. Prospective multicenter studies are needed to establish evidence-based diagnostic and management guidelines specific to primary intrapulmonary SFTs, an entity currently underrepresented in the literature and lacking dedicated management frameworks.
